# Hereditary Breast Cancer in the Brazilian State of Ceará (The CHANCE Cohort): Higher-Than-Expected Prevalence of Recurrent Germline Pathogenic Variants

**DOI:** 10.3389/fonc.2022.932957

**Published:** 2022-07-22

**Authors:** Ana Carolina Leite Vieira Costa Gifoni, Markus Andret Cavalcante Gifoni, Camila Martins Wotroba, Edenir Inez Palmero, Eduardo Leite Vieira Costa, Wellington dos Santos, Maria Isabel Achatz

**Affiliations:** ^1^ D’Or Institute for Research and Education (IDOR) and Rede D’Or São Carlos Hospital, Fortaleza, Brazil; ^2^ Surgery Department - Federal University of Ceara, Fortaleza, Brazil; ^3^ Cancer Research Center - Rede D'Or São Carlos Hospital, Fortaleza, Brazil; ^4^ Tumor Genetics Program - Brazilian National Cancer Institute (INCA) - Rio de Janeiro, Brazil and Molecular Oncology Research Center- Barretos Cancer Hospital, Barretos, Brazil; ^5^ Instituto de Ensino e Pesquisa Hospital Sirio Libanes and Instituto do Coração, HCFMUSP, São Paulo, Brazil; ^6^ Molecular Oncology Research Center- Barretos Cancer Hospital, Barretos, Brazil; ^7^ Oncology Center - Sirio Libanes Hospital, São Paulo, Brazil

**Keywords:** hereditary cancer, breast cancer, panel testing, BRCA1/2, PALB2, CHEK2, ATM, Northeast Brazil

## Abstract

**Purpose:**

There is a significant lack of epidemiological data on hereditary cancer in Northeast Brazil. This is the largest study on the prevalence and mutational spectrum of cancer predisposition genes conducted in this region and the first in the State of Ceará.

**Methods:**

Patients ≥18 years of age that were referred to CHANCE (Grupo de Câncer Hereditário do Ceará) from March 2014 to December 2020 with testing criteria for breast cancer susceptibility genes according to NCCN v.1.2021 were eligible to participate. The inclusion of patients was limited to one individual per family and to those born in the State of Ceará. All patients underwent a hereditary cancer panel testing with at least 30 genes.

**Results:**

A total of 355 patients were included, and 97 (27.3%) carried a P/LP germline variant in 18 different genes. Among the 97 P/LP carriers, *BRCA1* (31, 31.9%) and *BRCA2* (25, 25.7%) were the most frequently mutated genes, followed by *PALB2* (10, 10.3%), *CHEK2* (7, 7.2%) and *ATM* (4, 4.1%). A small number of recurrent variants (detected in three or more individuals) in *BRCA1*, *BRCA2*, *CHEK2* and *ATM* represented the majority of the P/LP variants described in this cohort.

**Conclusion:**

In this cohort, the prevalence of L/PL was high, particularly involving the *BRCA1*, *BRCA2*, *PALB2*, *CHEK2* and *ATM* genes and, to a lesser extent than expected, the *TP53* gene. A high frequency of recurrent variants was also observed, for which further and larger analyses should clarify the presence of any possible founder effect. Characterizing the mutational profile of cancer predisposition genes in diverse populations may contribute to cancer prevention and therapeutic management.

## Introduction

Hereditary pathogenic variants in cancer predisposition genes account for 7 to 10% of breast cancer cases (1, 2). The recognition of these patients and families provides important benefits in multiple aspects of patient’s care, including more intensive surveillance approaches, risk-reducing surgical strategies and targeted cancer therapies (3).

Hereditary Breast and Ovarian Cancer Syndrome (HBOC), the most prevalent breast cancer predisposition syndrome, is a highly penetrant autosomal dominant disorder caused by pathogenic germline variants in *BRCA1/2* genes. Although breast and ovarian cancer are the core tumors in HBOC (4), *BRCA1/2* mutations are also associated with other malignancies such as fallopian tube, peritoneal (5), prostate (6) and pancreatic cancer (7). Clinical management of HBOC has long been established, with solid benefits of risk reducing strategies especially related to bilateral salpingo-oophorectomy (8).

Other high and moderate penetrance genes included in multigene panels have been identified as predisposing to breast and ovarian cancer. Variants in at least 6 of these - *PALB2, ATM, CHEK2, BARD1, RAD51C, RAD51D* - are significantly associated with hereditary breast cancer (9, 10). More research is needed to determine the magnitude of cancer risk and the cancer spectrum of each of these genes (11). Moreover, further data will also help to clarify the value of surveillance and risk-reducing strategies and therefore current guidelines may change to accommodate new data as our understanding of these cancer predisposition syndromes expands.

It is increasingly clear that the knowledge of the population-specific mutational spectrum in cancer predisposition genes may contribute to better public policy choices and to improve cancer treatment. There is limited information about the epidemiology of hereditary cancer in Brazil (12), and most of the studies published to date come from the Southeast/South of the country (13). CHANCE (Grupo de Câncer Hereditário do Ceará) is a multidisciplinary group founded in 2013 as the first center of care, education and research on hereditary cancer in Ceará, one of the most populated Northeastern States in Brazil. The primary objective of the current study is to describe the epidemiological profile of hereditary breast cancer in the CHANCE cohort.

## Patients and Methods

### Study Population

Patients ≥18 years of age that were referred to CHANCE from March 2014 to December 2020 with testing criteria for breast cancer susceptibility genes according to National Comprehensive Cancer Network (NCCN) guidelines (version 1.2021) were eligible to participate. Informed consent for clinical testing was obtained for every patient. The inclusion of patients was limited to one individual per family and to those born in the State of Ceará. Clinical data, including age at cancer diagnosis and cancer family history, were obtained at pre-test genetic counseling evaluation. This information was anonymized and managed using REDCap electronic data capture tools. This study was approved by the local Ethics Committee.

### Procedures

Saliva or peripheral blood samples was collected and referred for molecular evaluation with an hereditary cancer panel at a College of American Pathology certified laboratory (mainly Genomika, Color Genomics and Invitae), chosen according to the financial possibilities or the health insurance coverage of each patient. The genes included in NGS panel varied by laboratory, ranging in number from 30 to 84 genes. In all cases, those definitely associated with hereditary predisposition to breast and gynecological cancer (*ATM, BARD1, BRCA1, BRCA2*, *BRIP1, CDH1, CHEK2, DICER1, EPCAM, MLH1, MSH2, MSH6, NF1, PALB2, PMS2, PTEN, RAD51C, RAD51D, STK11, TP53*) were analyzed.

### Statistical Analysis

Clinical characteristics of the CHANCE cohort, along with the molecular testing results, were tabulated. In order to explore the influence of distinct clinical scenarios on genetic test yield, which could lead to the recognition of priority criteria for molecular evaluation, this study also examined the association of specific NCCN criteria with a positive (P/LP variant) genetic test result. Descriptive statistics including medians, means and standard deviations with 95% confidence intervals was made by Mann Whitney (Wilcoxon rank sum) test. Categorical variables were analyzed by Fisher Exact or Chi-square test as appropriate. *P* values less than 0.05 were considered statistically significant. All statistical analyses were performed with the statistical programs R, version 4.1.0 (the CRAN project [http://cran.r-project.org]) and GraphPad Prism version 9.3.1 350.

## Results

### Study Population

A total of 736 probands born in the State of Ceará were referred to CHANCE for genetic counseling on hereditary cancer between 2014 and 2020 and of these, 516 (516/736 - 70.1%) met at least one NCCN testing criteria for breast and ovarian cancer susceptibility genes. Of this sample, 355 underwent genetic testing with hereditary cancer panel and were included in the study.

The majority of patients were female (94.2%) and 75,7% had a personal history of cancer, mainly breast (80%) and ovarian cancer (13%). The median age at the primary cancer diagnosis was 45 years (**Table 1**).

**Table 1:** Clinical characteristics of patients with breast or ovarian cancer in the CHANCE cohort.

Clinical Characteristics	N	%
**Index Patients**		
Breast Cancer	215	79.9
Ovarian Cancer	35	13.0
Breast and Ovarian Cancer	6	2.2
Breast Cancer and other	15	5.6
**Age at first diagnosis (years)**		
≤ 45	131	53.4
46-60	74	30.2
> 60	34	13.8
Not provided	6	2.6
**Sex**		
Female	253	94.0
Male	16	6.0
**Bilateral Breast Cancer**		
Yes	13	4.8
No	256	95.2
**Breast Cancer Subtype (IHC/receptor status)**		
ER+ PR+ HER2-	81	39.5
ER-, PR-, HER2+	4	2,0
Triple positive	13	6.3
Triple Negative	33	16.1
Not provided (any)	74	36.1
**First or second-degree relative with Breast or Ovarian Cancer**		
Yes	170	63.1
No	73	27.1
Not provided	26	9.8
**First or second-degree relative with any cancer**		
Yes	199	73.9
No	44	16.3
Not provided	26	9.8

### Genetic Testing Results

**Figure 1:**
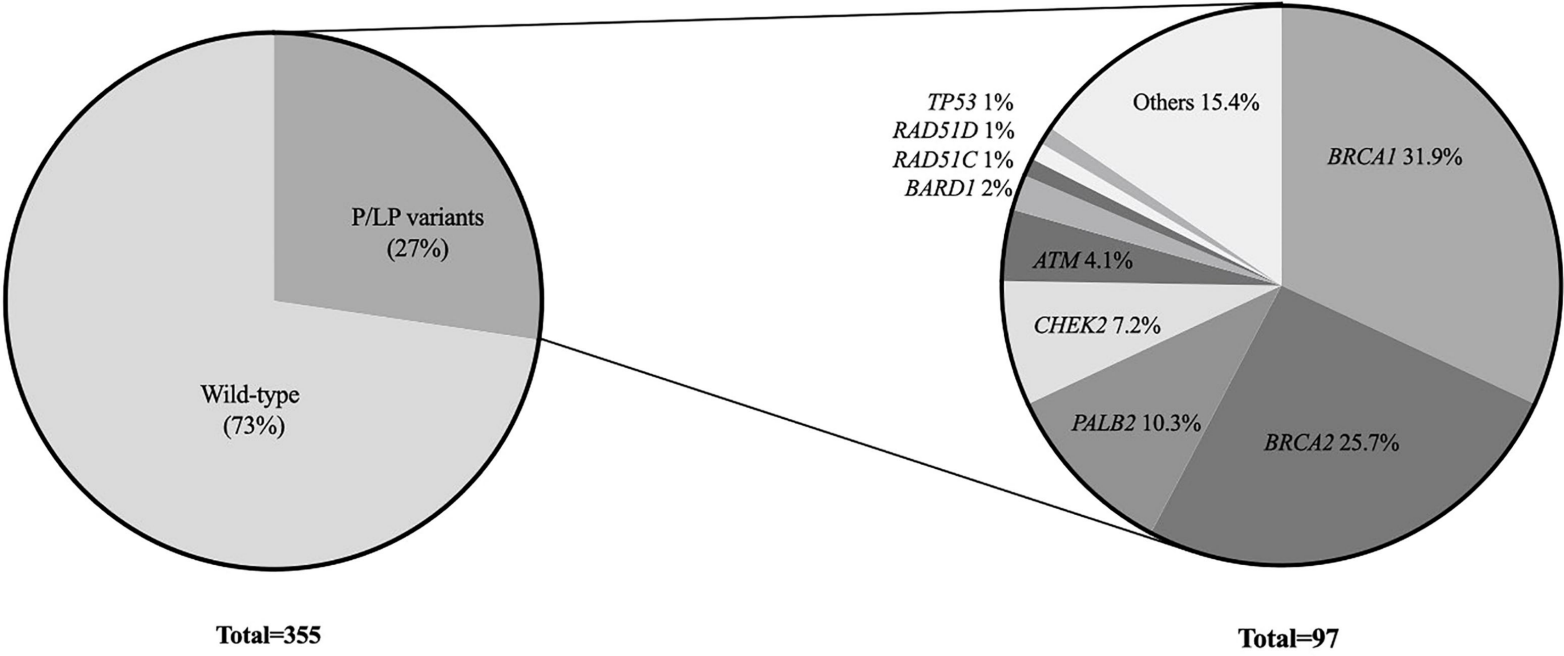
Oncoplot with P/LP variants: variants types and frequencies according to age at first diagnosis and primary tumor site.

Among all 355 patients, 97 (27.3%) carried a pathogenic or likely pathogenic (P/LP) germline variant in 18 different genes including high penetrant breast cancer genes - *BRCA1* (31, 8.7%), *BRCA2* (25, 7.0%), *PALB2* (10, 2.8%) and *TP53* (1, 0.28%); moderate-penetrant breast cancer genes - *CHEK2* (7, 1.9%), *ATM* (4, 1.1%), *BARD1* (2, 0.56%), *RAD51C* (1, 0.28%) and *RAD51D* (1, 0.28%); and genes without an established association with breast cancer predisposition (15, 4.2%): *PMS2* (4, 1.1%)*, MUTYH* (3, 0.8%), *MEN1* (2, 0.56%), *NTHL1* (1, 0.28%), *RAD50* (1, 0.28%), *SDHA* (1, 0.28%)*, SUFU* (1, 0.28%), *DICER1* (1, 0.28%) and *BLM1* (1, 0.28%) (**Figure 1**). The association between specific scenarios from the NCCN testing criteria and the detection of P/LP variants was not statistically significant for age, primary tumor type, or family history of cancer (**Table 2**).

**Table 2:** Association between personal and family criteria for genetic testing and the finding of P/LP germline variants.

	Number of Patients							
Clinical Criteria	Total Cohort					*BRCA1*		
	**Negative result**		**P/LP variant**	***P***		**Negative result**		**P/LP variant**
**N (Total)**	258		97			324		31
**Age ≤ 45 (%)**	134 (51.9)		52 (53.6)	0.872		168 (51.9)		18 (58.1)
**Type of Cancer**								
Breast Cancer (%)	154 (59.7)		61 (62.9)	0.669		194 (59.9)		21 (67.7)
Ovarian Cancer (%)	22 (8.5)		13 (13.4)	0.241		28 (8.6)		7 (22.6)
Breast and Ovarian Cancer (%)	2 (0.8)		4 (4.1)	0.086		4 (1.2)		2 (6.5)
Personal history of any cancer (%)	195 (75.6)		74 (76.3)	1.000		243 (75.0)		26 (83.9)
**Family History of Breast or Ovarian Cancer**				0.327				
No (%)	56 (21.7)		21 (21.6)			71 (21.9)		6 (19.4)
Not available (%)	17 (6.6)		11 (11.3)			22 (6.8)		6 (19.4)
Yes (%)	185 (71.7)		65 (67.0)			231 (71.3)		19 (61.3)
**Family History of Cancer**				0.154				
No (%)	29 (11.2)		15 (15.5)			36 (11.1)		8 (25.8)
Not available (%)	17 (6.6)		11 (11.3)			22 (6.8)		6 (19.4)
Yes (%)	212 (82.2)		71 (73.2)			266 (82.1)		17 (54.8)
	**(%)**							
**Clinical Criteria**		***BRCA2***						
	***P***	**Negative result**			**P/LP variant**		***P***	
**N (Total)**		330			25			
**Age ≤ 45 (%)**	0.636	169 (51.2)			17 (68.0)		0.158	
**Type of Cancer**								
Breast Cancer (%)	0.507	196 (59.4)			19 (76.0)		0.154	
Ovarian Cancer (%)	0.030	31 (9.4)			4 (16.0)		0.471	
Breast and Ovarian Cancer (%)	0.155	4 (1.2)			2 (8.0)		0.083	
Personal history of any cancer (%)	0.378	248 (75.2)			21 (84.0)		0.451	
**Family History of Breast or Ovarian Cancer**	0.046						0.263	
No (%)		73 (22.1)			4 (16.0)			
Not available (%)		24 (7.3)			4 (16.0)			
Yes (%)		233 (70.6)			17 (68.0)			
**Family History of Cancer**	0.010						0.294	
No (%)		41 (12.4)			3 (12.0)			
Not available (%)		24 (7.3)			4 (16.0)			
Yes (%)		265 (80.3)			18 (72.0)			

At least one variant of uncertain significance (VUS) was identified in 107 (30.1%) patients, with 20 having two VUS, 7 having three VUS and 2 having four VUS. The highest frequency of VUS was observed in *BRCA2* and *ATM*. None of the VUS was detected in more than two individuals in this cohort (**Supplementary Table 1**).

### Mutational Profile

**Figure 2:**
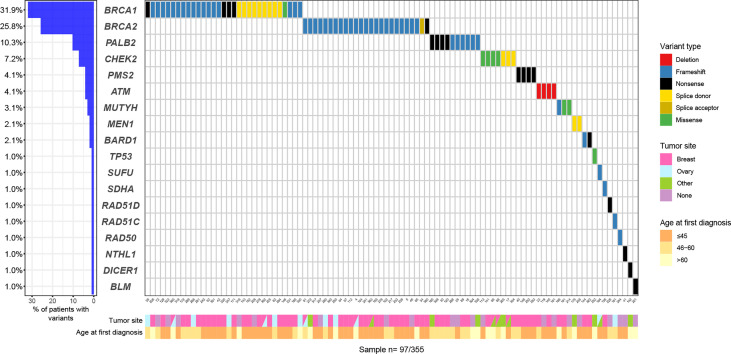
P/LP variants per gene in CHANCE cohort.

Among the 97 P/LP carriers, *BRCA1* (31, 31.9%), *BRCA2* (25, 25.7%) were the most frequently mutated genes, followed by *PALB2* (10, 10.3%), *CHEK2* (7, 7.2%) and *ATM* (4, 4.1%). The mutational profile is shown in **Figure 2**.

**Figure 3:**
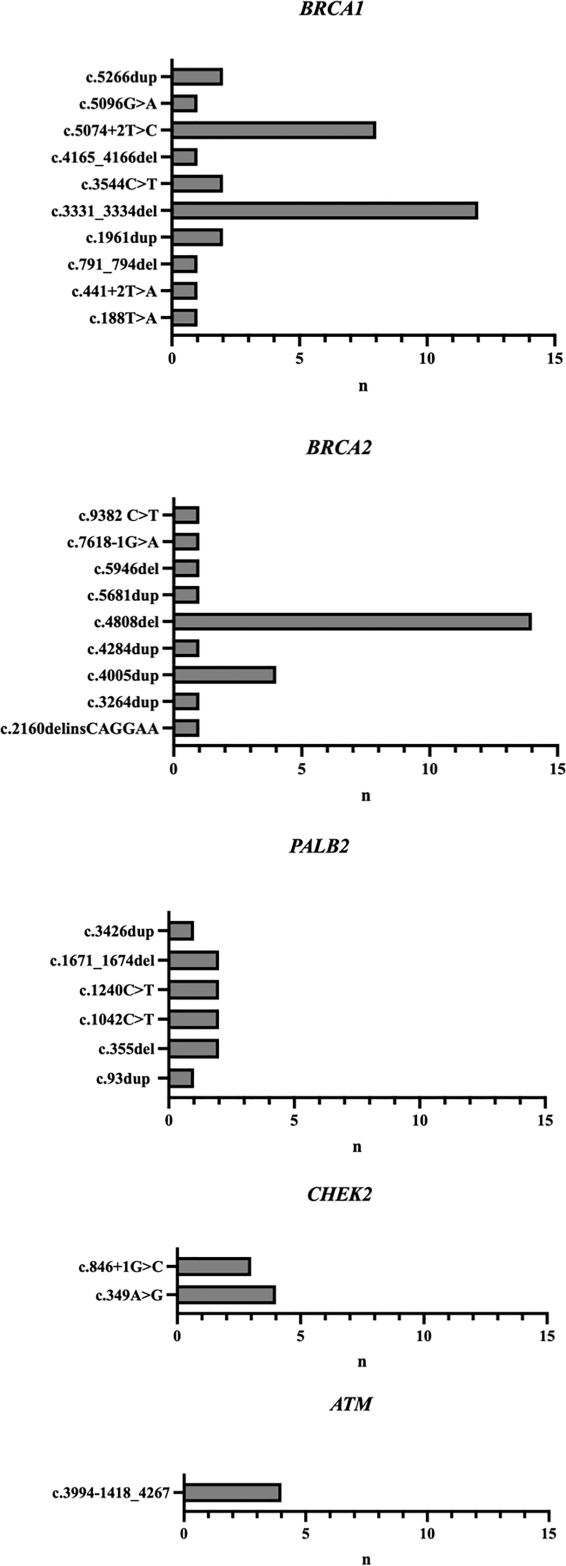
P/LP variants in five most frequently mutated genes.

A small number of recurrent variants (detected in three or more individuals) in *BRCA1*, *BRCA2*, *CHEK2* and *ATM* represented the majority of the P/LP variants described in this cohort (**Figure 3**). Two recurrent variants accounted for 64.5% of all P/LP variants reported in *BRCA1*, the frameshift alteration c.3331_3334del in exon 11 representing 38.7% (n=12) and c.5074+2T>C corresponding to 25.8% (n=8). Among carriers of *BRCA2* P/LP variants, the recurrent variant c.4808del was identified in 56% (n=14) and c.4005dup in 16% (n=4) of the cases. The Europeanfounder *CHEK2* recurrent variant c.349A>G accounted for 66.6% (n=4) of the P/LP *CHEK2* variants, and the other 33.3% corresponded to another recurrent variant, c.846+1G>C (n=3). All carriers of P/LP variants in the *ATM* gene (n=4) had the same partial deletion encompassing exons 27-29.

Of note, the only patient with Li-Fraumeni syndrome in this cohort had the c.743G>C variant in the *TP53* gene. The Brazilian variant R337H was not described in this cohort.

## Discussion

### Study Population

This is the largest study that reports on the spectrum and frequency of P/LP variants in breast cancer patients who underwent multigene panel in the State of Ceará, Brazil. The most valuable contribution of this study is the suggestion of a distinctive genetic architecture of hereditary predisposition to breast cancer in the State of Ceará compared to the rest of the country. Notably, it points to a very relevant role of recurrent variants in genes such as *BRCA1*, *BRCA2*, *CHEK2* and *ATM* and a minor contribution of the Brazilian founder variant R337H in *TP53* gene.

The prevalence of P/LP variants in this cohort was 27.3% (97/355), higher than that described in the most recent studies using multigene panel testing in the Brazilian population. Recent data from diverse regions of the country estimated a detection rate of P/LP variants of 16.9% in the Northern (14); 20.8% in the Northeast (15), 20.5% in the Central-west (16) and 23.4% in Southern Brazil (17). Also, in the largest nationwide cohort of Brazilian breast cancer patients, the prevalence of germline P/LP variants was 20.1% (18). Unlike the studies cited, many with consecutive samples, our cohort has the bias of representing only high-risk patients who met NCCN criteria for genetic testing. Thus, the patient selection may account for the higher prevalence of P/LP variants in this cohort, although some previous data question the ability of the NCCN guidelines to identify a breast cancer population enriched with pathogenic variants in expanded panel testing (19). In this cohort, there was no statistically significant association between any specific NCCN criteria scenarios and the detection of a P/LP variant. This suggests that the individual weights of each criterion appear to be equivalent, and that the sum of one or more criteria does not seem to predict a greater chance of a positive result.

### Genetic Testing Results

Pathogenic germline variants in this cohort were distributed among 18 different genes, with a relevant contribution of actionable non-*BRCA* genes, suggesting that molecular investigation in Brazilian patients fulfilling NCCN criteria should preferably be done with multigene panel testing. Sixty-nine percent (67/97) of the patients with a positive test result harbored a P/LP in high-penetrance breast cancer genes (32% in *BRCA1*, 25.7% in *BRCA2*, 10.3% in *PALB2* and 1% in *TP53*) and 15.4% (15/97) in moderate-penetrant breast cancer genes (7.2% in *CHEK2*, 4.1% in *ATM*, 2% in *BARD1*, 1% in *RAD51C* and 1% in *RAD51D*). Overall, 57.7% (56/97) of the patients with a positive test result had *BRCA1/2* P/LP variants and 21% (26/97) had variants in non-*BRCA* genes. Cancer risks and management approaches are well-defined for women with P/LP in high penetrance cancer syndrome genes, especially for *BRCA1/2* and *TP53*, enabling early diagnosis and improved overall survival (3, 20, 21). For genes that confer modest degree of risk, the best practices, including timing and effectiveness of interventions, although incompletely defined, are addressed in internacional guidelines (22).

The predominance of *BRCA1/2* P/LP variants in this study is in agreement with the literature. Also in accordance with previous published data, the three most frequently mutated non-*BRCA1/2* genes in this study were *PALB2*, *CHEK2* and *ATM* (2, 10). However, more recent Brazilian data described a distinct distribution of P/LP variants, with a much more relevant prevalence of variants in *TP53* and *MUTYH* genes than identified in this cohort. In the nationwide study, *TP53* was the third most frequently mutated gene (10.5% of P/LP variants), and *MUTYH* the fourth (9.9% of P/LP variants) (18). Although the nationwide analysis includes patients from all Brazilian regions, more than half of the tests were from patients who inhabited the Southeast region of Brazil. The results of the present study may reflect the high degree of genetic admixture among Brazilian populations and highlight the importance of the epidemiological description of cancer predisposition syndromes in specific populations.

At the same time that the findings from this study pointed to multigene panel as the preferential method of assessing inherited risk in this population, it also raised challenges that reinforce the critical role of genetic counseling in this process. First, P/LP variants in genes without an established association with breast cancer predisposition were found in 15.4% of the patients with a positive test result (4.1% in *PMS2*, 3% in *MUTYH*, 2% in *MEN1*, and 1% in *NTHL1, RAD50*, *SDHA, SUFU*, *DICER1* and *BLM1*), equivalent to 4.2% of the overall study population. In these scenarios, genetic counseling should attempt to identify in which clinical situations these variants are just incidental findings, when they should be meaningful and how they should interfere in personalized management of the patient and the family. In addition, the frequency of VUS in this study was 30.1%, similar to that expected for panels with at least 30 genes (23). In these situations, genetic counseling seeks to interpret the molecular finding in the context of the personal and family phenotype, and plays an essential role in avoiding inappropriate management.

### Mutational Profile

The most relevant finding in the mutational profile of this cohort was the high prevalence of recurrent variants in *BRCA1*, *BRCA2*, *ATM* and *CHEK2*, a pattern that justifies further investigation of possible founder effects. Also noteworthy was the absence of the Brazilian variant R337H in the *TP53* gene.

In *BRCA1*, there were two recurrent variants in this study (c.3331_3334del and c.5074+2T>C). The most prevalent one was c.3331_3334del, corresponding to 38.7% of the *BRCA1* P/LP variants. This frameshift alteration was previously described as the second most commonly identified pathogenic *BRCA1* variant in Brazil (13), was originated in Iberia and was later introduced to Colombia and South America at the time of Spanish colonization (24). The second most prevalent *BRCA1* P/LP variant described in this cohort was c.5074+2T>C, corresponding to 25.8% of the cases. In the largest comprehensive description of the spectrum of germline *BRCA* mutations in Brazil, c.5074+2T>C was detected in 14/441 individuals with *BRCA1* mutations (3,1%) (13). In the recently published nationwide cohort, this variant was not considered recurrent (18). Thus, when compared to the rest of the country, this cohort of patients from the state of Ceará seems to be enriched of this variant. In contrast, the founder Ashkenazi Jew pathogenic variant c.5266dupC, considered the most frequent *BRCA1* variant in the Brazilian population by several studies and representing 20% of the *BRCA1* variants reported by Palmero etal. (13), was identified in only 2 individuals in this study, corresponding to 6.4% of the P/LP *BRCA1* variants. The small representation of this variant in the population of this study, from the Northeast Brazil, may be related to the fact that the immigration of Ashkenazi Jews from Central Europe during the 19th century occurred particularly to the Southeast and South regions of the country, where the mutation is nowadays more frequently described (25, 26).

In *BRCA2*, there was a high prevalence of the recurrent variant c.4808delA, accounting for 56% of all *BRCA2* P/LP variants. Previous studies have not identified such a significant role for a specific variant in the *BRCA2* gene. In Palmero’s study, with 208 individuals with P/LP mutations in *BRCA2*, 103 different mutations were described. The most prevalent was the variant c.2808_2811delACAA, reaching 9.6% of cases, and the c.4808del variant accounted for only 1.4% of *BRCA2* P/LP variants. The c.4808del variant was also not relevant in other Brazilian studies. (16–18, 27). Also, in the CIMBA (Consortium of Investigators of Modifiers of *BRCA1/2*) publication that assembled data on 29,700 families with *BRCA1/2* mutations from 49 countries, c.4808delA was not included in the group of the 10 most frequently observed *BRCA2* mutations from any race/ethnicity or from any of the 6 continents (28). The differential burden of the c.4808del variant in *BRCA2* gene in the state of Ceará is likely due to differences in structure across populations but a deeper explanation of its origin and distribution remains uncharacterized.

In *CHEK2*, two recurrent P/LP variants were identified (c.349A>G and c.846+1G>C). The most frequent one, c.349A>G (57.1%, 4/7), well described in the European population, is considered recurrent in Portugal (29). The variant c.846+1G>C was described in 3/7 patients from this study (42.8%). Both variants were previously reported in a small number in Brazil (16).

In *ATM*, all 4 patients shared the variant c.3994-1418_4267, also knows as Deletion (Exons 27-29), for which the prevalence and geographical distribution in populations has not yet been well studied.

In Brazil, there is a higher than the global incidence of Li-Fraumeni syndrome due to the presence of the founder mutation in the *TP53* gene (30), c.1010G>A;p.Arg337His (p.R337H), which occurs at a frequency of 0.3% in the South and Southeast of the country (31). This high prevalence is consistently seen in studies of hereditary predisposition to breast cancer. Recently, *TP53* represented the third most commonly mutated gene among breast cancer Brazilian patients, and the R337H variant was responsible for 70.3% of all P/LP *TP53* variants identified (18). According to the results of the present study, Li-Fraumeni syndrome seems to play a smaller role in cancer predisposition in the State of Ceará, and the Brazilian founder *TP53* variant R337H was not identified in any patients from this cohort. There is a hypothesis that the R337H allele was introduced in Brazil by Portuguese immigrants in the first four decades of the 1700s, progressing along transit routes that became known as the “Tropeiro roads” (32). Possibly, the low prevalence of R337H in this cohort is related to the significant distance between Ceará and this geographical area.

## Conclusion

In summary, to our knowledge, this is the largest study to describe the epidemiological profile of hereditary breast cancer in patients from the State of Ceará with criteria for hereditary predisposition to cancer and undergoing investigation with a multigene panel. In this cohort, the prevalence of L/PL was high, particularly involving the *BRCA1*, *BRCA2*, *PALB2*, *CHEK2* and *ATM* genes and, to a lesser extent than expected, the *TP53* gene. A high frequency of recurrent variants was also observed, for which further and larger analyses should clarify the presence of any possible founder effect.

This study reinforces the importance of characterizing the mutational profile of cancer predisposition genes in diverse populations.

## Data Availability Statement

‘The original contributions presented in the study are included in the article/**Supplementary Materials**. Further inquiries can be directed to the corresponding author.

## Ethics Statement

The studies involving human participants were reviewed and approved by São Carlos Hospital Ethics Committee (Plataforma Brasil Protocol n. 4.474.595). The patients/participants provided their written informed consent to participate in this study.

## Author Contributions

Conceptualization: AG and MA. Data curation: AG, MG and CW. Formal analysis: AG, MG and EC. Investigation: AG and CW. Methodology: AG, MG, CW, MA, EC, EP, and WdS. Supervision: AG and MA. Writing: AG, MG, and EC. All authors contributed to the article and approved the submitted version.

## Conflict of Interest

The authors declare that the research was conducted in the absence of any commercial or financial relationships that could be construed as a potential conflict of interest.

## Publisher’s Note

All claims expressed in this article are solely those of the authors and do not necessarily represent those of their affiliated organizations, or those of the publisher, the editors and the reviewers. Any product that may be evaluated in this article, or claim that may be made by its manufacturer, is not guaranteed or endorsed by the publisher.
