# Supplementary material for: Hereditary Breast Cancer in the Brazilian State of Ceará (The CHANCE Cohort): Higher-Than-Expected Prevalence of Recurrent Germline Pathogenic Variants
Source: Front Oncol. 2022 Jul 22;12:932957. doi: 10.3389/fonc.2022.932957 (PMC9361024; doi:10.3389/fonc.2022.932957)
Supplement: Supplementary file 1 [file DataSheet_1.pdf]

ID	Sex Female or Male	Result	Gene	Description (MANE SELECT)	Variant type	Molecular consequence	Personal Cancer Diagnosis Yes (Affected); No (Not Affected)	Breast Cancer Yes; No	Bilateral Cancer Yes; No	Ovarian Cancer Yes; No	Breast and Ovarian Cancer Yes; No	Breast and Other Cancer Yes; No	Age at First Cancer Diagnosis (Breas or Ovarian) ≤ 45; 46–60; > 60; Not available	Other cancer Yes (which one); No	Subtypes, Receptor Status ER+, PR+, HER2− ER+, PR-, HER2− ER+, PR−, HER2+ ER−, PR−, HER2+ Triple positive Triple negative Not available	First/Second degree family member with breast or ovarian cancer Yes; No; Not available	PFirst/Second degree family member with any cancer Yes; No; Not available	Genetic Testing
1	Female	Negative	NA	NA			Yes	Yes	No	No	No	No	≤ 45	No	ER+, RP+, Her2-	Yes	Yes	Panel
2	Female	VUS	BMPR1A	c.1520A>G p.(Asn507Ser)			Yes	Yes	No	No	No	No	≤45	No	CDIS	Yes	Yes	Panel
3	Female	Negative	NA	NA			Yes	Yes	No	No	No	No	≤ 45	No	ER+, PR-, HER2−	Yes	Yes	Panel
4	Female	Positive	BRCA2	c.4808del	Deletion	Frameshift	Yes	Yes	No	Yes	Yes	No	> 60	No	ER+, PR+, HER2−	Not available	Not available	Panel
5	Female	Positive	BRCA2	c.4808del	Deletion	Frameshift	Yes	Yes	No	No	No	No	≤ 45	No	ER+, PR -, HER2−	No	No	Panel
6	Female	VUS	CASR<br>TERT	c.856C>T (p.Arg286Cys)<br>c.1336C>G (p.Arg446Gly)			Yes	Yes	No	Yes	Yes	Yes	46–60	GIST	ER+, PR+, Her2-	No	Yes	Panel
7	Female	VUS	MUTYH	c.1309C>T (p.Arg437Trp)			Yes	Yes	No	No	No	No	≤ 45	No	ER+, PR+, HER2−	Yes	Yes	Panel
8	Female	VUS	APC	c.6782C>T p.(Pro2261Leu)			Yes	Yes	No	No	No	No	≤ 45	No	ER+, PR-, HER2−	No	Yes	Panel
9	Female	VUS	ATM	Crom 11 Posição<br>108160506 Variação T>G<br>c.4424 T>G (anotação)			Yes	Yes	No	No	No	No	≤ 45	No	Triple negative	No	No	Panel
10	Male	Negative	NA	NA			Yes	No	No	No	No	No	≤45	Kidney	NA	Yes	No	Panel
11	Female	Negative	NA	NA			No	No	No	No	No	No	≤45	No	NA	Yes	Yes	Panel
12	Female	VUS	GATA2	c.707T>C (p.Met236Thr)			Yes	Yes	Yes	No	No	No	≤ 45	No	Triple positive<br>ER−, PR−, HER2+	Yes	Yes	Panel
13	Female	Negative	NA	NA			Yes	Yes	No	No	No	No	46–60	No	ER−, PR−, HER2+	Yes	No	Panel
14	Female	Negative	NA	NA			Yes	Yes	No	No	No	No	≤ 45	No	ER+, PR+, Her2-	Yes	Yes	Panel
15	Female	VUS	BRCA2<br>BRCA2	c.1148T>A:p.I383N<br>c.4187A>G:p.Q1396R			Yes	Yes	No	No	No	No	46–60	No	Not available	Yes	Yes	Panel
16	Female	Negative	NA	NA			Yes	Yes	No	No	No	No	≤ 45	No	ER+, PR+, HER2−	No	No	Panel
17	Female	Positive	CHEK2	c.846+1G>C	Single nucleotid	Splice donor	Yes	Yes	No	No	No	Yes	≤ 45	Apendice	ER+, PR+, Her2-	No	Yes	Panel
18	Female	Positive	PALB2	c.93dup	Duplication	Frameshift	Yes	Yes	No	No	No	No	46–60	No	ER+, PR+, Her2-	Yes	Yes	Panel
19	Female	Negative	NA	NA			No	No	No	No	No	No	46-60	NA	NA	Yes	Yes	Panel
20	Female	VUS	TSC2	c.2048C>A (p.Ser683Tyr)			Yes	Yes	No	No	No	No	46–60	No	ER+, PR+, Her2-	Yes	Yes	Panel
21	Female	VUS	APC	c.1580G>C (p.Arg527Thr)			Yes	No	No	Yes	No	No	> 60	No	NA	Not available	Not available	Panel
22	Female	Negative	NA	NA			No	No	No	No	No	No	46-60	NA	NA	Yes	Yes	Panel
23	Female	Positive	ATM	c.3994-1418_4267	Deletion		Yes	Yes	No	No	No	No	46–60	No	ER+, PR+, HER2−	No	Yes	Panel
24	Female	VUS	ATM	Crom 11 Posição			Yes	Yes	No	No	No	No	≤ 45	No	Triple negative	Yes	Yes	Panel
25	Female	Negative	NA	NA			Yes	Yes	No	No	No	No	≤ 45	No	Triple positive	No	Yes	Panel
26	Female	Positive	PALB2	c.1671_1674del	Microsatellite	Frameshift	No	No	No	No	No	No	≤45	No	NA	Yes	Yes	Panel
27	Female	VUS	FLCN	c.65C>T (p.Thr22Met)			Yes	Yes	No	No	No	No	≤ 45	No	Triple positive	Yes	Yes	Panel
28	Female	Negative	NA	NA			Yes	Yes	No	No	No	No	> 60	No	Triple negative	Not available	Not available	Panel
29	Female	Negative	NA	NA			Yes	Yes	Yes	No	No	No	≤ 45	No	ER+, PR−, HER2+	Yes	Yes	Panel
30	Female	Positive	PALB2	c.1240C>T	Single nucleotid	Nonsense	Yes	Yes	No	No	No	No	46–60	No	ER+, PR+, Her2-	Yes	Yes	Panel
31	Female	Negative	NA	NA			Yes	No	No	No	No	No	46–60	Melanoma	NA	Yes	Yes	Panel
32	Male	VUS	ATM	c.7375C>G (p.Arg2459Gly)			No	No	No	No	No	No	>60	NA	NA	No	Yes	Panel
33	Male	VUS	NF1	c.100G>A (p.Val34Ile)			Yes	No	No	No	No	No	≤45	CRC	NA	Yes	Yes	Panel
34	Female	Positive	BRCA2	c.7618-1G>A	Single nucleotid	Splice acceptor	Yes	Yes	No	No	No	No	≤ 45	No	Triplo Negative	No	Yes	Panel
35	Female	VUS	BRCA2	c.G5704A:p.D1902N			No	No	No	No	No	No	≤45	No	NA	Yes	Yes	Panel
36	Female	VUS	BRIP1	c.1066C>G (p.Arg356Gly)			No	No	No	No	No	No	≤45	No	NA	Yes	Yes	Panel
37	Male	Negative	NA	NA			No	No	No	No	No	No	46-60	No	NA	Yes	Yes	Panel
38	Female	VUS	CASP8	c.35A>C p.E12A			Yes	Yes	No	Yes	Yes	No	46–60	No	CDIS	Yes	Yes	Panel
39	Female	Positive	BRCA1	c.188T>A	Single nucleotid	Nonsense	Yes	No	No	Yes	No	No	46–60	No	NA	No	No	Panel
40	Female	Positive	PMS2	c.631C>T	Single nucleotid	Nonsense	Yes	Yes	No	No	No	No	46–60	No	ER+, PR+, HER2−	No	No	Panel
41	Female	Positive	NTHL1	c.268C>T	Single nucleotid	Nonsense	No	No	No	No	No	No	≤45	No	NA	Yes	Yes	Panel
42	Female	Positive	BRCA1	c.3331_3334	Deletion	Frameshift	Yes	Yes	No	No	No	No	≤ 45	No	Triplo Negative	No	No	Panel
43	Female	Negative	NA	NA			No	No	No	No	No	No	≤45	No	NA	Yes	Yes	Panel
44	Female	Negative	NA	NA			Yes	Yes	No	No	No	No	46–60	No	Triplo Negative	Yes	Yes	Panel

ID	Sex Female or Male	Result	Gene	Description (MANE SELECT)	Variant type	Molecular consequence	Personal Cancer Diagnosis Yes (Affected); No (Not Affected)	Breast Cancer Yes; No	Bilateral Cancer Yes; No	Ovarian Cancer Yes; No	Breast and Ovarian Cancer Yes; No	Breast and Other Cancer Yes; No	Age at First Cancer Diagnosis (Breas or Ovarian) ≤ 45; 46–60; > 60; Not available	Other cancer Yes (which one); No	Subtypes, Receptor Status<br>ER+, PR+, HER2−<br>ER+, PR-, HER2−<br>ER+, PR−, HER2+<br>ER−, PR−, HER2+<br>Triple positive<br>Triple negative<br>Not available	First/Second degree family member with breast or ovarian cancer Yes; No; Not available	PFirst/Second degree family member with any cancer Yes; No; Not available	Genetic Testing
45	Female	Negative	NA	NA			No	No	No	No	No	No	≤45	No	NA	Yes	Yes	Panel
46	Female	Positive	BRCA2	c.5681dup	Duplication	Frameshift	No	No	No	No	No	No	≤45	No	NA	Yes	Yes	Panel
47	Female	Negative	NA	NA			Yes	Yes	No	No	No	No	> 60	No	Triplo Negative	Yes	No	Panel
48	Female	VUS	PDGFRA	c.1325T>C p. (Leu442Pro)			Yes	No	No	Yes	No	No	> 60	No	NA	Not available	Not available	Panel
49	Female	Negative	NA	NA			Yes	Yes	No	No	No	No	46–60	No	CDIS	Yes	No	Panel
50	Female	VUS	ATM	c.1049C>T (p.Ala350Val)			No	No	No	No	No	No	> 60	No	NA	Yes	Yes	Panel
51	Female	VUS	MUTYH	c.650G>A (p.Arg217His)			Yes	No	No	Yes	No	No	> 60	No	NA	No	Yes	Panel
52	Female	Negative	NA	NA			Yes	Yes	No	No	No	No	≤ 45	No	NA	Yes	Yes	Panel
53	Female	Negative	NA	NA			No	No	No	No	No	No	46-60	No	NA	No	Yes	Panel
54	Female	VUS	CDKN1B	c.349C>T (p.Pro117Ser)			No	No	No	No	No	No	>60	No	NA	Yes	Yes	Panel
55	Female	VUS	MEN1	c.883C>T (p.Arg295Trp)			No	No	No	No	No	No	46-60	No	NA	Not available	Not available	Panel
56	Female	Negative	NA	NA			No	No	No	No	No	No	≤45	No	NA	Yes	Yes	Panel
57	Female	Negative	NA	NA			No	No	No	No	No	No	≤45	No	NA	No	Yes	Panel
58	Female	Negative	NA	NA			No	No	No	No	No	No	≤45	No	NA	Yes	Yes	Panel
59	Female	Negative	NA	NA			Yes	Yes	No	No	No	No	≤ 45	No	ER+, RP+, Her2-	Not available	Not available	Panel
60	Female	Negative	NA	NA			Yes	Yes	No	No	No	No	≤ 45	No	Not available	Yes	Yes	Panel
61	Female	VUS	MSH3	c.205C>G (p.Pro69Ala)			Yes	No	No	Yes	No	Yes	≤ 45	Thyroid	NA	Yes	Yes	Panel
62	Female	Positive	BRCA1	c.5074+2T>C	Single nucleotid	Splice donor	Yes	No	No	Yes	No	No	46–60	No	NA	Not available	Not available	Panel
63	Female	Negative	NA	NA			No	No	No	No	No	No	>60	No	NA	Yes	Yes	Panel
64	Female	Positive	BRCA2	c.4808del	Deletion	Frameshift	Yes	Yes	No	No	No	No	≤ 45	No	Triplo Negative	Yes	Yes	Panel
65	Male	Positive	BRCA2	c.5946del	Deletion	Frameshift	Yes	Yes	No	No	No	No	> 60	No	Triplo Positive	No	Yes	Panel
66	Female	VUS	APC	c.8524T>G (p.Ser2842Ala)			No	No	No	No	No	No	46-60	No	NA	Yes	Yes	Panel
67	Female	Negative	NA	NA			Yes	Yes	No	No	No	No	≤ 45	No	ER+, PR+, HER2−	Yes	Yes	Panel
68	Female	Negative	NA	NA			Yes	Yes	No	No	No	No	≤ 45	No	ER+, PR+, HER2−	Yes	Yes	Panel
69	Female	VUS	BRCA2	c.7676C>G (p.Ser2559Cys)			No	No	No	No	No	No	≤45	No	NA	Yes	Yes	Panel
70	Female	Negative	NA	NA			No	No	No	No	No	No	≤45	No	NA	Yes	Yes	Panel
71	Female	Negative	NA	NA			Yes	Yes	No	No	No	No	≤ 45	No	Triple negative	Not available	Not available	Panel
72	Female	Positive	BRCA1	c.1961dup	Duplication	Frameshift	No	No	No	No	No	No	46-60	No	NA	Not available	Not available	Panel
73	Female	Negative	NA	NA			No	No	No	No	No	No	≤45	No	NA	Yes	Yes	Panel
74	Female	VUS	ATM	c.4091A>G (p.Asp1364Gly)			Yes	Yes	No	No	No	No	≤ 45	No	Not available	No	Yes	Panel
75	Female	Negative	NA	NA			Yes	Yes	No	No	No	No	≤ 45	No	ER+, PR+, HER2−	Yes	Yes	Panel
76	Male	VUS	BARD1	c.1835A>T (p.Asp612Val)			Yes	Yes	No	No	No	No	> 60	No	ER+, PR+, HER2−	Yes	No	Panel
77	Female	Negative	NA	NA			Yes	Yes	Yes	No	No	No	46–60	No	ER+, PR+, HER2−	Yes	Yes	Panel
78	Female	VUS	ATM	c.7816A>G (p.Ile2606Val)			Yes	No	No	Yes	No	No	46–60	No	NA	No	Yes	Panel
79	Female	Negative	NA	NA			Yes	No	No	Yes	No	No	46–60	No	NA	Yes	Yes	Panel
80	Female	Negative	NA	NA			Yes	Yes	No	No	No	No	46-60	No	Not available	Not available	Not available	Panel
81	Female	Positive	BRCA2	c.2163delinsCAGGAA	Delins		Yes	Yes	No	Yes	Yes	No	46-60	No	Not available	Not available	Not available	Panel
82	Female	Negative	NA	NA			Yes	Yes	No	No	No	No	46–60	No	ER+, PR+, HER2−	Yes	No	Panel
83	Female	Negative	NA	NA			Yes	Yes	No	No	No	No	46–60	No	ER+, PR+, HER2−	Not available	Not available	Panel
84	Female	VUS	DIS3L2	c.1687G>A (p.Glu563Lys)			Yes	No	No	No	No	No	46-60	No	NA	No	Yes	Panel
85	Female	VUS	EGFR	c.2885G>A (p.Arg962His)			Yes	Yes	No	No	No	No	≤ 45	Prostate	Not available	Yes	Yes	Panel
86	Female	Positive	CHEK2	c.349A>G	Single	Missense	Yes	Yes	No	No	No	No	>60	Endometrial	Not available	Not available	Not available	Panel
87	Female	Negative	NA				Yes	Yes	No	No	No	No	46–60	No	Not available	Yes	Yes	Panel
88	Female	Positive	CHEK2	c.349A>G	Single	Missense	Yes	Yes	No	No	No	Yes	46–60	Thyroid		Yes	Yes	Panel
89	Female	Positive	PALB2	c.355del	Deletion	Frameshift	Yes	Yes	No	No	No	No	46–60	No	ER+, PR+, HER2−	Yes	Yes	Panel
90	Female	Negative	NA	NA			Yes	Yes	No	No	No	No	≤ 45	NA	ER+, PR+, HER2−	Not available	Not available	Panel
91	Female	VUS	BLM	c.543C>A (p.Ser181Arg)			Yes	No	No	No	No	No	≤45	Kidney	NA	Yes	Yes	Panel
92	Female	Positive	BRCA1	c.3331_3334	Deletion	Frameshift	Yes	Yes	No	No	No	No	≤45	No	Not available	No	Yes	Panel

ID	Sex Female or Male	Result	Gene	Description (MANE SELECT)	Variant type	Molecular consequence	Personal Cancer Diagnosis Yes (Affected); No (Not Affected)	Breast Cancer Yes; No	Bilateral Cancer Yes; No	Ovarian Cancer Yes; No	Breast and Ovarian Cancer Yes; No	Breast and Other Cancer Yes; No	Age at First Cancer Diagnosis (Breas or Ovarian) ≤ 45; 46–60; > 60; Not available	Other cancer Yes (which one); No	Subtypes, Receptor Status ER+, PR+, HER2− ER+, PR-, HER2− ER+, PR−, HER2+ ER−, PR−, HER2+ Triple positive Triple negative Not available	First/Second degree family member with breast or ovarian cancer Yes; No; Not available	PFirst/Second degree family member with any cancer Yes; No; Not available	Genetic Testing
93	Female	VUS	DIS3L2	c.2227C>T (p.Arg743Cys)			Yes	Yes	No	No	No	No	46–60	No	Not available	Yes	No	Panel
94	Female	Negative	NA	NA			Yes	Yes	No	No	No	No	≤ 45	No	Not available	No	Yes	Panel
95	Female	Negative	NA	NA			No	No	No	No	No	No	≤45	No	NA	Yes	Yes	Panel
96	Female	Negative	NA	NA			No	No	No	No	No	No	≤45	No	NA	Yes	Yes	Panel
97	Female	Positive	BRCA2	c.4808del	Deletion	Frameshift	Yes	Yes	No	No	No	No	≤45	No	Triplo Negative	Yes	Yes	Panel
98	Female	Negative	NA	NA			Yes	Yes	No	No	No	No	46–60	No	ER+, PR+, HER2−	No	No	Panel
99	Female	VUS	ATM	c.5753G>C (p.Arg1918Thr)			Yes	Yes	No	No	No	No	≤ 45	No	ER+, PR+, HER2−	No	Yes	Panel
100	Female	Negative	NA	NA			Yes	Yes	No	No	No	No	46–60	No	ER+, PR+, HER2−	Yes	Yes	Panel
101	Female	VUS	BRIP1	c.797C>T (p.Thr266Met)			Yes	Yes	No	No	No	No	≤ 45	No	Triplo Negative	Yes	Yes	Panel
102	Female	Positive	BRCA2	c.4808del	Deletion	Frameshift	No	No	No	No	No	No	46-60	No	NA	Yes	Yes	Panel
103	Female	Negative	NA	NA			Yes	Yes	No	No	No	No	≤ 45	No	Not available	Yes	Yes	Panel
104	Female	Positive	BRCA2	c.4808del	Deletion	Frameshift	Yes	Yes	No	No	No	No	≤ 45	No	Triplo Negative	Yes	Yes	Panel
105	Female	Negative	NA	NA			Yes	Yes	No	No	No	No	46–60	No	Not available	Yes	Yes	Panel
106	Female	Negative	NA	NA			Yes	No	No	Yes	No	No	46–60	No	NA	Yes	Yes	Panel
107	Female	Negative	NA	NA			No	No	No	No	No	No	≤45	No	NA	Yes	Yes	Panel
108	Female	Negative	NA	NA			Yes	No	No	No	No	No	>60	Gastric	NA	Yes	Yes	Panel
109	Female	Negative	NA	NA			Yes	Yes	No	No	No	No	≤ 45	No	ER−, PR−, HER2+	No	No	Panel
110	Female	VUS	MSH2	c.703A>G p.(Lys235Glu)			Yes	Yes	No	No	No	No	≤ 45	No	Not available	Yes	Yes	Panel
111	Female	Negative	NA	NA			Yes	Yes	No	No	No	No	> 60	No	Triple negative	Yes	Yes	Panel
112	Female	Positive	BRCA2	c.4808del	Deletion	Frameshift	Yes	Yes	No	No	No	No	≤ 45	No	ER+, PR+, HER2−	Yes	Yes	Panel
113	Female	Positive	CHEK2	c.349A>G	Single	Missense	No	No	No	No	No	No	≤45	No	NA	Yes	Yes	Panel
114	Female	VUS	BLM	c.543C>A (p.Ser181Arg)			No	No	No	No	No	No	46-60	No	NA	Yes	Yes	Panel
115	Male	VUS	BRCA2	c.2401A>G (p.Asn801Asp)			Yes	No	No	No	No	No	>60	Prostate	NA	Yes	Yes	Panel
116	Female	VUS	PDGFRA	c.3216G>T (p.Met1072Ile)			Yes	Yes	No	No	No	Yes	≤ 45	Thyroid	Not available	No	No	Panel
117	Female	Negative	NA	NA			Yes	Yes	No	No	No	No	≤ 45	No	Not available	Yes	Yes	Panel
118	Female	VUS	BRCA2	c.1483G>A p.Ala495Thr			Yes	Yes	No	No	No	No	≤ 45	No	ER+, PR+, HER2−	No	No	Panel
119	Female	Positive	ATM	c.3994-1418_4267	Deletion		No	No	No	No	No	No	≤45	No	NA	Yes	Yes	Panel
120	Female	Positive	PALB2	c.1240C>T	Single nucleotid	Nonsense	Yes	Yes	No	No	No	No	46–60	No	ER+, PR+, HER2−	Yes	No	Panel
121	Female	Negative	NA	NA			Yes	Yes	No	No	No	No	≤ 45	No	ER+, PR+, HER2−	No	Yes	Panel
122	Female	Negative	NA	NA			Yes	Yes	No	No	No	No	> 60	No	ER+, PR+, Her2 -	Yes	Yes	Panel
123	Female	Negative	NA	NA			Yes	Yes	No	No	No	No	≤ 45	No	Not available	Yes	Yes	Panel
124	Female	VUS	MSH2	c.314A>G (p.Asn105Ser)			Yes	No	No	No	No	No	46-60	Skin	NA	Yes	Yes	Panel
125	Female	Negative	NA	NA			Yes	Yes	No	No	No	No	≤ 45	No	Not available	Yes	Yes	Panel
126	Female	Negative	NA	NA			Yes	Yes	No	No	No	No	> 60	No	Triple negative	No	Yes	Panel
127	Female	Negative	NA	NA			Yes	Yes	No	No	No	No	≤ 45	NA	ER+, PR+, HER2−	Not available	Not available	Panel
128	Female	VUS	BRCA1	c.1012A>G (p.Lys338Glu)			Yes	Yes	Yes	No	No	No	46–60	No	ER+, PR+, HER2−	Yes	Yes	Panel
129	Female	Positive	BRCA1	c.3331_3334	Deletion	Frameshift	Yes	Yes	No	No	No	No	≤ 45	No	Triplo Negative	No	No	Panel
130	Female	Negative	NA	NA			Yes	No	No	Yes	No	No	≤ 45	No	NA	No	Yes	Panel
131	Female	VUS	CTNNA1	c.710A>G (p.Tyr237Cys)			Yes	Yes	No	No	No	No	≤ 45	No	ER+, PR+, HER2−	No	No	Panel
132	Female	VUS	TSC2	c.3779C>T (p.Thr1260Met)			Yes	Yes	No	No	No	Yes	46–60	Thyroid	ER+, PR+, HER2−	Yes	Yes	Panel
133	Female	VUS	BLM	c.1601A>G (p.Asn534Ser)			Yes	Yes	No	No	No	No	≤ 45	No	ER+, PR+, HER2−	Not available	Not available	Panel
134	Female	VUS	BRCA1	c.1724A>G:p.E575G			Yes	Yes	No	No	No	Yes	≤ 45	Thyroid	ER+, PR+, HER2−	No	No	Panel
135	Female	VUS	BRCA2	c.7A>G: p.I3V			No	No	No	No	No	No	≤45	No	NA	Yes	Yes	Panel
136	Female	Positive	SDHA	c.554dup	Duplication	Frameshift	Yes	Yes	No	No	No	No	≤ 45	No	ER+, PR+, HER2−	Yes	Yes	Panel
137	Female	Negative	NA	NA			Yes	Yes	No	No	No	No	≤ 45	No	ER+, PR+, HER2−	Yes	Yes	Panel
138	Male	Negative	NA	NA			Yes	No	No	No	No	No	>60	Pancreas	NA	No	Yes	Panel
139	Female	Negative	NA	NA			Yes	No	No	Yes	No	No	≤ 45	No	NA	No	Yes	Panel
140	Male	Negative	NA	NA			Yes	Yes	No	No	No	No	46–60	No	Not available	Yes	Yes	Panel

ID	Sex Female or Male	Result	Gene	Description (MANE SELECT)	Variant type	Molecular consequence	Personal Cancer Diagnosis Yes (Affected); No (Not Affected)	Breast Cancer Yes; No	Bilateral Cancer Yes; No	Ovarian Cancer Yes; No	Breast and Ovarian Cancer Yes; No	Breast and Other Cancer Yes; No	Age at First Cancer Diagnosis (Breas or Ovarian) ≤ 45; 46–60; > 60; Not available	Other cancer Yes (which one); No	Subtypes, Receptor Status ER+, PR+, HER2− ER+, PR-, HER2− ER−, PR−, HER2+ ER−, PR−, HER2+ Triple positive Triple negative Not available	First/Second degree family member with breast or ovarian cancer Yes; No; Not available	PFirst/Second degree family member with any cancer Yes; No; Not available	Genetic Testing
141	Male	Positive	CHEK2	c.349A>G	Single	Missense	Yes	Yes	No	No	No	No	> 60	No	ER+, PR+, HER2−	No	Yes	Panel
142	Male	Positive	DICER1	c.4252G>T (p.Glu1418*)	Single nucleotid	Nonsense	Yes	No	No	No	No	No	>60	Prostate	NA	No	Yes	Panel
143	Female	Positive	BRCA1	c.5074+2T>C	Single nucleotid	Splice donor	Yes	Yes	Yes	No	No	No	≤45	No	ER+, PR+, HER2−	Not available	Not available	Panel
144	Female	Positive	BARD1	c.176_177del	Deletion	Frameshift	Yes	Yes	No	No	No	No	≤ 45	No	Not available	No	Yes	Panel
145	Female	Positive	ATM	c.3994-1418_4267	Deletion		Yes	Yes	No	No	No	No	≤ 45	No	ER+, PR+, HER2−	No	Yes	Panel
146	Female	Positive	BRCA1	c.5096G>A	Single nucleotid	Missense	Yes	Yes	No	No	No	No	≤45	No	ER+, PR+, Her2 -	No	No	Panel
147	Female	Negative	NA	NA			No	No	No	No	No	No	≤45	No	NA	Not available	Not available	Panel
148	Female	Positive	PMS2	c.631C>T	Single nucleotid	Nonsense	Yes	Yes	No	No	No	No	≤ 45	No	ER+, PR+, HER2−	Yes	Yes	Panel
149	Female	Negative	NA	NA			Yes	Yes	No	No	No	No	≤ 45	No	Not available	No	Yes	Panel
150	Female	Negative	BRCA1	c.1067G: p.Q356R			No	No	No	No	No	No	46-60	No	NA	Yes	Yes	Panel
151	Female	VUS	CHEK2	c.1141A>G (p.Met381Val)			Yes	Yes	No	No	No	No	≤ 45	No	ER+, PR+, HER2−	Yes	Yes	Panel
152	Female	Positive	BRCA1	c.5074+2T>C	Single nucleotid	Splice donor	Yes	Yes	No	No	No	No	≤ 45	No	Triple negative	Yes	Yes	Panel
153	Female	VUS	CHEK2	c.1130A>G p.(Glu377Gly)			Yes	Yes	No	No	No	No	≤ 45	No	Triple negative	No	Yes	Panel
154	Female	VUS	SDHB	c.112C>T (p.Arg38Cys)			No	No	No	No	No	No	≤45	No	NA	Yes	Yes	Panel
155	Female	Positive	BRCA2	c.4808del	Deletion	Frameshift	Yes	Yes	No	No	No	No	≤ 45	No	ER+, PR+, HER2−	Yes	No	Panel
156	Female	Negative	NA	NA			Yes	Yes	No	No	No	No	46–60	No	Not available	Yes	Yes	Panel
157	Female	VUS	ALK	c.3254C>A (p.Thr1085Asn)			Yes	Yes	No	No	No	Yes	≤ 45	Linfoma de H	ER−, PR−	Not available	Not available	Panel
158	Female	Negative	NA	NA			Yes	Yes	No	No	No	No	≤ 45	No	ER+, PR+, HER2−	Yes	No	Panel
159	Female	VUS	AIP	c.965C>T (p.Ala322Val)			Yes	Yes	No	No	No	No	≤ 45	No	Triple negative	Yes	Yes	Panel
160	Female	VUS	ATM	c.6998C>T (p.Thr2333Ile)			Yes	Yes	No	No	No	No	≤ 45	No	Triple negative	Yes	Yes	Panel
161	Female	Positive	ATM	c.3994-1418_4267	Deletion		No	No	No	No	No	No	≤45	No	NA	Yes	Yes	Panel
162	Female	Negative	NA	NA			No	No	No	No	No	No	≤45	No	NA	Yes	Yes	Panel
163	Male	Negative	NA	NA			Yes	No	No	No	No	No	≤45	CCR	NA	Yes	Yes	Panel
164	Female	Positive	SUFU	c.71dup	Duplication	Frameshift	Yes	No	No	Yes	No	No	≤45	Meningioma Meduloblast oma Thyroid	NA	Yes	Yes	Panel
165	Female	Positive	PALB2	c.1042C>T	Single nucleotid	Nonsense	No	No	No	No	No	No	46-60	CCR	NA	Yes	Yes	Panel
166	Female	Negative	NA	NA			Yes	Yes	No	No	No	No	46–60	No	ER+, PR+, HER2−	Yes	Yes	Panel
167	Female	Negative	NA	NA			Yes	Yes	No	No	No	No	46–60	No	CDIS RE 100%	Yes	Yes	Panel
168	Female	Negative	NA	NA			Yes	Yes	No	No	No	No	46–60	No	HER2+	Yes	Yes	Panel
169	Female	Negative	NA	NA			Yes	Yes	No	No	No	No	≤ 45	No	ER+, PR+, HER2−	Yes	Yes	Panel
170	Female	VUS	AXIN2	c.1577A>G (p.Lys526Arg)			Yes	Yes	No	No	No	No	46–60	No	ER+, PR-, HER2−	No	Yes	Panel
171	Female	Positive	BRCA1	c.4165_4166del	Microsatellite	Nonsense	Yes	Yes	No	No	No	No	≤ 45	No	Triplo Negative	Not available	Not available	Panel
172	Female	Positive	BRCA1	c.3331_3334	Deletion	Frameshift	Yes	Yes	No	No	No	No	46-60	No	Not available	Not available	Not available	Panel
173	Female	Negative	NA	NA			Yes	Yes	No	No	No	No	≤ 45	No	Triplo Negative	No	No	Panel
174	Male	Negative	NA	NA			Yes	No	No	No	No	No	>60	Prostata	NA	No	Yes	Panel
175	Female	Positive	MEN1	c.669+1G>T	Single nucleotide variant		Yes	No	No	No	No	No	>60	Pancreas	NA	No	Yes	Panel
176	Female	Negative	NA	NA			Yes	Yes	No	No	No	No	46–60	No	Not available	Yes	Yes	Panel
177	Female	VUS	BARD1	c.1835A>T (p.Asp612Val)			Yes	Yes	No	No	No	No	46–60	No	ER+, PR+, HER2−	Yes	Yes	Panel
178	Female	Negative	NA	NA			Yes	No	No	Yes	No	No	≤ 45	No	NA	No	Yes	Panel
179	Female	Negative	NA	NA			Yes	Yes	Yes	No	No	No	46–60	No	Not available	Not available	Not available	Panel
180	Female	Negative	NA	NA			Yes	Yes	No	No	No	No	>60	No	Not available	Not available	Not available	Panel
181	Female	Positive	RAD51C	c.890_899del	Deletion	Frameshift	Yes	No	No	Yes	No	No	46–60	No	NA	Yes	Yes	Panel
182	Female	Negative	NA	NA			Yes	Yes	Yes	No	No	Yes	46–60	Leiomiosarc	Not available	Not available	Not available	Panel
183	Female	Negative	NA	NA			No	No	No	No	No	No	46-60	No	NA	Yes	Yes	Panel
184	Female	Negative	NA	NA			Yes	No	No	Yes	No	No	>60	No	NA	Yes	Yes	Panel
185	Female	Positive	BRCA1	c.5266dup	Duplication	Duplication	Yes	Yes	No	No	No	No	46-60	No	Not available	Yes	No	Panel
186	Female	Positive	MUTYH	c.1147del	Deletion	Frameshift	Yes	Yes	No	No	No	No	> 60	No	Not available	Yes	Yes	Panel

ID	Sex Female or Male	Result	Gene	Description (MANE SELECT)	Variant type	Molecular consequence	Personal Cancer Diagnosis Yes (Affected); No (Not Affected)	Breast Cancer Yes; No	Bilateral Cancer Yes; No	Ovarian Cancer Yes; No	Breast and Ovarian Cancer Yes; No	Breast and Other Cancer Yes; No	Age at First Cancer Diagnosis (Breas or Ovarian) ≤ 45; 46–60; > 60; Not available	Other cancer Yes (which one); No	Subtypes, Receptor Status ER+, PR+, HER2− ER+, PR-, HER2− ER+, PR−, HER2+ ER−, PR−, HER2+ Triple positive Triple negative Not available	First/Second degree family member with breast or ovarian cancer Yes; No; Not available	PFirst/Second degree family member with any cancer Yes; No; Not available	Genetic Testing
187	Female	Negative	NA	NA			Yes	Yes	Yes	No	No	No	> 60	No	Not available	Yes	Yes	Panel
188	Female	VUS	BRCA1	c.5643C>G: p.H1881Q			Yes	Yes	No	No	No	No	≤ 45	No	ER+, PR+, Her2-	No	No	Panel
189	Female	Negative	NA	NA			Yes	Yes	No	No	No	No	46-60	Thyroid	NA	Yes	Yes	Panel
190	Female	VUS	POLE	c.158T>C (p.Leu53Pro)			Yes	Yes	No	No	No	Yes	≤ 45	Thyroid	Not available	No	Yes	Panel
191	Female	Positive	MUTYH	c.1103G>A	Single nucleotid	Missense	Yes	Yes	Yes	No	No	No	≤ 45	No	Not available	Yes	Yes	Panel
192	Female	Negative	NA	NA			Yes	No	No	Yes	No	No	≤ 45	No	NA	Yes	No	Panel
193	Female	VUS	BARD1	c.2206T>C (p.Tyr736His)			Yes	Yes	No	No	No	Yes	46-60	Lung	ER+, PR-, HER2−	No	No	Panel
194	Female	VUS	PTCH1	c.4202A>G (p.Tyr1401Cys)			Yes	No	No	No	No	No	46-60	Kidney	NA	Yes	Yes	Panel
195	Female	VUS	POLE	c.-6G>C			Yes	Yes	No	No	No	No	≤ 45	No	Not available	Yes	Yes	Panel
196	Female	Negative	NA	NA			Yes	Yes	No	No	No	No	>60	No	ER+, PR+, HER2−	No	Yes	Panel
197	Female	Negative	NA	NA			Yes	Yes	No	No	No	No	>60	No	Not available	Yes	Yes	Panel
198	Female	Negative	NA	NA			Yes	No	No	Yes	No	No	>60	No	NA	Yes	Yes	Panel
199	Female	Negative	NA	NA			Yes	Yes	No	No	No	No	46-60	No	Not available	Yes	Yes	Panel
200	Female	Positive	BRCA1	c.3544C>T	Single nucleotid	Nonsense	Yes	Yes	No	No	No	No	≤45	No	Not available	Yes	Yes	Panel
201	Female	Positive	BLM	c.2268del	Deletion	Nonsense	No	No	No	No	No	No	>60	No	NA	Yes	Yes	Panel
202	Female	Negative	NA	NA			Yes	Yes	No	No	No	No	>60	No	Inconclusivo	Yes	Yes	Panel
203	Female	Negative	NA	NA			Yes	No	No	Yes	No	No	>60	No	NA	No	No	Panel
204	Female	Negative	NA	NA			Yes	No	No	No	No	No	>60	Pancreas	NA	Yes	Yes	Panel
205	Female	Positive	CHEK2	c.846+1G>C	Single nucleotid	Splice donor	Yes	No	No	No	No	No	>60	Pancreas	NA	No	No	Panel
206	Female	Positive	BRCA1	c.1961dup	Duplication	Frameshift	Yes	Yes	No	No	No	No	> 60	No	Triple negative	Yes	Yes	Panel
207	Female	Negative	NA	NA			No	No	No	No	No	No	≤45	No	NA	Yes	Yes	Panel
208	Female	VUS	BLM	c.1216A>G (p.Ile406Val)			No	No	No	No	No	No	≤45	No	NA	Yes	Yes	Panel
209	Female	Positive	BRCA1	c.5074+2T>C	Single nucleotid	Splice donor	No	No	No	No	No	No	≤45	No	NA	Yes	Yes	Panel
210	Female	Negative	NA	NA			Yes	Yes	No	No	No	No	> 60	No	ER+, PR-, HER2−	Yes	Yes	Panel
211	Female	Negative	NA	NA			Yes	No	No	Yes	No	No	>60	No	NA	Not available	Not available	Panel
212	Female	VUS	MSH6	NA			Yes	Yes	No	No	No	No	≤ 45	No	ER+, PR+, HER2−	No	Yes	Panel
213	Female	Negative	NA	NA			No	No	No	No	No	No	≤45	No	NA	Yes	Yes	Panel
214	Female	Positive	MUTYH	c.1103G>A	Single nucleotid	Missense	No	No	No	No	No	No	46-60	No	NA	Yes	Yes	Panel
215	Female	Negative	NA	NA			Yes	Yes	No	No	No	No	46-60	No	Triple positive	Yes	Yes	Panel
216	Female	Negative	NA	NA			Yes	Yes	No	No	No	No	≤ 45	No	Not available	No	Yes	Panel
217	Female	Positive	BRCA2	c.4808del	Deletion	Frameshift	Yes	Yes	No	No	No	No	46-60	No	ER+, PR-, HER2−	Yes	Yes	Panel
218	Female	Negative	NA	NA			Yes	Yes	No	No	No	No	≤ 45	CBC	Not available	Yes	Yes	Panel
219	Female	VUS	BRCA2	c.A1114C: p.N372H			Yes	Yes	No	No	No	No	≤ 45	No	Triple negative	Yes	Yes	Panel
220	Female	Negative	NA	NA			Yes	Yes	No	No	No	No	≤ 45	No	Not available	No	Yes	Panel
221	Female	Negative	NA	NA			Yes	Yes	No	No	No	No	≤ 45	No	Not available	Yes	Yes	Panel
222	Female	Negative	NA	NA			Yes	Yes	No	No	No	No	46-60	No	ER+, PR+, HER2−	Yes	Yes	Panel
223	Female	Negative	BRCA1	c.A1067G :p.Q356R			No	No	No	No	No	No	≤45	No	NA	Yes	Yes	Panel
224	Female	VUS	POLE	c.3857G>A p.(Arg1286His)			No	No	No	No	No	No	46-60	No	NA	Yes	Yes	Panel
225	Female	Positive	BRCA1	c.5074+2T>C	Single nucleotid	Splice donor	Yes	Yes	Yes	No	No	No	≤45	No	ER+, PR+, Her2 -	Yes	Yes	Panel
226	Female	Positive	RAD51D	c.694C>T	Single nucleotid	Nonsense	No	No	No	No	No	No	46-60	No	NA	Yes	Yes	Panel
227	Female	Negative	NA	NA			Yes	Yes	No	No	No	No	≤ 45	No	NA	Yes	Yes	Panel
228	Female	VUS	PDGFRA	c.1438G>A (p.Asp480Asn)			No	No	No	No	No	No	≤45	No	NA	Yes	Yes	Panel
229	Female	Negative	NA	NA			No	No	No	No	No	No	≤45	No	NA	Yes	Yes	Panel
230	Female	Negative	NA	NA			Yes	Yes	No	No	No	No	≤ 45	No	Triple positive	No	No	Panel
231	Female	Positive	BRCA1	c.5266dup	Duplication	Duplication	Yes	Yes	No	No	No	No	≤45	No	Not available	Yes	Yes	Panel
232	Female	VUS	BRCA2	c.1148T>A(ILe383Asn)			Yes	No	No	Yes	No	Yes	>60	Thyroid	NA	No	Yes	Panel
233	Female	Positive	BRCA2	c.4808del	Deletion	Frameshift	Yes	Yes	No	No	No	No	≤ 45	No	Triple positive	Not available	Not available	Panel
234	Female	VUS	TSC2	c.2048C>A (p.Ser683Tyr)			Yes	No	No	No	No	No	>60	Pancreas	NA	Yes	Yes	Panel

ID	Sex Female or Male	Result	Gene	Description (MANE SELECT)	Variant type	Molecular consequence	Personal Cancer Diagnosis Yes (Affected); No (Not Affected)	Breast Cancer Yes; No	Bilateral Cancer Yes; No	Ovarian Cancer Yes; No	Breast and Ovarian Cancer Yes; No	Breast and Other Cancer Yes; No	Age at First Cancer Diagnosis (Breas or Ovarian) ≤ 45; 46–60; > 60; Not available	Other cancer Yes (which one); No	Subtypes, Receptor Status ER+, PR+, HER2− ER+, PR-, HER2− ER+, PR−, HER2+ ER−, PR−, HER2+ Triple positive Triple negative Not available	First/Second degree family member with breast or ovarian cancer Yes; No; Not available	PFirst/Second degree family member with any cancer Yes; No; Not available	Genetic Testing
**235**	Male	Positive	MEN1	c.669+1G>T	Single nucleotide variant		No	No	No	No	No	No	≤45	No	NA	Yes	Yes	Panel
**236**	Female	VUS	DIS3L2	c.1447C>G (p.Arg483Gly)			Yes	Yes	No	No	No	No	≤ 45	No	ER+, PR-, HER2−	Yes	Yes	Panel
**237**	Female	Positive	BRCA2	c.4005dup	Duplication	Frameshift	No	No	No	No	No	No	≤45	No	NA	Yes	Yes	Panel
**238**	Male	VUS	BRCA1	c.4097-141A>C			No	No	No	No	No	No	>60	No	NA	Yes	Yes	Panel
**239**	Female	Positive	BRCA2	c.4808del	Deletion	Frameshift	Yes	Yes	No	No	No	No	≤45	No	Not available	Not available	Not available	Panel
**240**	Female	Negative	NA	NA			Yes	No	No	Yes	No	No	≤ 45	No	NA	Yes	Yes	Panel
**241**	Female	Positive	BRCA2	c.4808del	Deletion	Frameshift	Yes	Yes	No	No	No	No	≤ 45	No	ER+, PR+, HER2−	Yes	No	Panel
**242**	Female	Positive	BRCA1	c.3331_3334	Deletion	Frameshift	Yes	Yes	No	No	No	No	≤ 45	No	Triplo Negative	Yes	No	Panel
**243**	Female	Negative	NA	NA			No	No	No	No	No	No	≤45	Apendix	NA	Yes	Yes	Panel
**244**	Female	Positive	RAD50	c.2517dup	Duplication	Frameshift	No	No	No	No	No	No	≤45	No	NA	Yes	Yes	Panel
**245**	Female	Negative	NA	NA			Yes	Yes	Yes	No	No	No	≤ 45	No	Not available	Yes	Yes	Panel
**246**	Female	Negative	NA	NA			No	No	No	No	No	No	46-60	No	NA	Yes	Yes	Panel
**247**	Female	Positive	BRCA1	c.3544C>T	Single nucleotid	Nonsense	Yes	No	No	Yes	No	No	46-60	No	NA	Not available	Not available	Panel
**248**	Female	Negative	NA	NA			Yes	Yes	No	No	No	No	46-60	No	ER+, PR+, HER2−	Yes	Yes	Panel
**249**	Female	Negative	NA	NA			No	No	No	No	No	No	>60	No	NA	Yes	Yes	Panel
**250**	Female	Positive	BRCA2	c.4284dup	Duplication	Frameshift	Yes	No	No	Yes	No	No	>60	No	NA	No	Yes	Panel
**251**	Female	Negative	NA	NA			Yes	Yes	No	No	No	No	≤ 45	No	NA	Yes	Yes	Panel
**252**	Male	Positive	TP53	c.743G>C	Single nucleotid	Missense	Yes	No	No	No	No	No	>60	LLC	NA	Yes	Yes	Panel
**253**	Female	Positive	PMS2	c.631C>T	Single nucleotid	Nonsense	Yes	Yes	No	No	No	No	≤ 45	No	Not available	Yes	Yes	Panel
**254**	Female	Negative	NA	NA			No	No	No	No	No	No	≤45	No	NA	Yes	Yes	Panel
**255**	Female	VUS	HRAS	c.290+6C>A (Intronic)			No	No	No	No	No	No	≤45	No	NA	Yes	Yes	Panel
**256**	Female	VUS	BRCA2	c.9976A>T (p.K3326X)			Yes	Yes	No	No	No	No	≤ 45	No	Not available	Yes	Yes	Panel
**257**	Female	VUS	RAD51C	c.904G>A (p.Gly302Arg)			Yes	No	No	Yes	No	No	46-60	No	NA	Yes	Yes	Panel
**258**	Female	VUS	APC	Crom: 5 Posição:			Yes	Yes	No	No	No	No	≤ 45	No	ER−, PR−, HER2+	No	Yes	Panel
**259**	Female	VUS	APC	c.-30366C>T (Non-coding)			Yes	Yes	No	No	No	No	≤ 45	No	ER+, PR+, HER2−	No	Yes	Panel
**260**	Female	Negative	NA	NA			Yes	Yes	No	No	No	No	≤ 45	No	ER+, PR+, HER2−	No	Yes	Panel
**261**	Male	Negative	NA	NA			Yes	No	No	No	No	No	>60	Kidney	NA	Yes	Yes	Panel
**262**	Female	Negative	NA	NA			Yes	Yes	No	No	No	No	46-60	No	Not available	Yes	Yes	Panel
**263**	Female	Positive	PMS2	c.631C>T	Single nucleotid	Nonsense	Yes	Yes	No	No	No	No	≤ 45	No	Not available	Yes	Yes	Panel
**264**	Female	Positive	BRCA1	c.5074+2T>C	Single nucleotid	Splice donor	Yes	Yes	No	No	No	No	≤ 45	No	Triplo Negative	No	Yes	Panel
**265**	Female	VUS	POLD1	c.353C>T (p.Ser118Phe)			Yes	No	No	Yes	No	No	> 60	No	NA	Yes	Yes	Panel
**266**	Female	Positive	BRCA1	c.3331_3334	Deletion	Frameshift	Yes	No	No	Yes	No	No	45-60	No	NA	Yes	Yes	Panel
**267**	Female	VUS	CDKN1C	c.389A>T (p.Glu130Val)			Yes	Yes	No	No	No	No	≤ 45	No	Not available	Yes	Yes	Panel
**268**	Female	Positive	PALB2	c.1671_1674del	Microsatellite	Frameshift	No	No	No	No	No	No	45-60	No	NA	Yes	Yes	Panel
**269**	Female	Negative	NA	NA			Yes	Yes	No	No	No	No	≤ 45	No	Not available	Yes	No	Panel
**270**	Female	Negative	NA	NA			Yes	Yes	No	No	No	No	>60	No	Not available	Yes	Yes	Panel
**271**	Female	Negative	NA	NA			Yes	Yes	No	No	No	No	>60	No	ER+, PR+, HER2−	Yes	Yes	Panel
**272**	Male	Positive	BRCA2	c.3264dup	Duplication	Frameshift	No	No	No	No	No	No	>60	Gastric	NA	Yes	Yes	Panel
**273**	Female	VUS	APC	c.6637A>G (p.Met2213Val)			No	No	No	No	No	No	46-60	No	NA	Yes	Yes	Panel
**274**	Female	Negative	NA	NA			Yes	Yes	No	No	No	No	46-60	No	Not available	Yes	Yes	Panel
**275**	Female	VUS	BRCA2	c.5270A>G (p.Tyr1757Cys)			No	No	No	No	No	No	46-60	No	NA	Yes	Yes	Panel
**276**	Female	Positive	BRCA2	c.4808del	Deletion	Frameshift	Yes	Yes	No	No	No	No	≤ 45	No	Not available	Yes	Yes	Panel
**277**	Female	Negative	NA	NA			No	No	No	No	No	No	≤45	No	NA	Yes	Yes	Panel
**278**	Female	Positive	BRCA1	c.5074+2T>C	Single nucleotid	Splice donor	Yes	Yes	No	No	No	No	≤ 45	No	Not available	Yes	Yes	Panel
**279**	Female	Negative	NA	NA			No	No	No	No	No	No	46-60	No	NA	Yes	Yes	Panel
**280**	Female	Positive	BRCA2	c.4005dup	Duplication	Frameshift	Yes	No	No	Yes	No	No	46-60	No	NA	Yes	Yes	Panel
**281**	Female	Positive	BRCA1	c.3331_3334	Deletion	Frameshift	Yes	Yes	No	No	No	No	46-60	No	Triple positive	Yes	No	Panel
**282**	Female	VUS	PMS2				Yes	No	No	No	No	No	46-60	Pancreas	NA	No	No	Panel

ID	Sex Female or Male	Result	Gene	Description (MANE SELECT)	Variant type	Molecular consequence	Personal Cancer Diagnosis Yes (Affected); No (Not Affected)	Breast Cancer Yes; No	Bilateral Cancer Yes; No	Ovarian Cancer Yes; No	Breast and Ovarian Cancer Yes; No	Breast and Other Cancer Yes; No	Age at First Cancer Diagnosis (Breas or Ovarian) ≤ 45; 46–60; > 60; Not available	Other cancer Yes (which one); No	Subtypes, Receptor Status ER+, PR+, HER2− ER+, PR-, HER2− ER+, PR−, HER2+ ER−, PR−, HER2+ Triple positive Triple negative Not available	First/Second degree family member with breast or ovarian cancer Yes; No; Not available	PFirst/Second degree family member with any cancer Yes; No; Not available	Genetic Testing
283	Female	Negative	NA	NA			Yes	Yes	No	No	No	No	46-60	CBC	Not available	Yes	No	Panel
284	Female	Negative	NA	NA			Yes	Yes	No	No	No	No	≤ 45	No	Triple positive	No	Yes	Panel
285	Female	Negative	NA	NA			No	No	No	No	No	No	≤45	No	NA	Yes	Yes	Panel
286	Female	VUS	ATM	c.4148C>T (p.Ser1383Leu)			Yes	Yes	No	No	No	No	46-60	No	ER+, PR+, HER2−	Yes	Yes	Panel
287	Female	Negative	NA	NA			Yes	Yes	No	No	No	No	≤ 45	No	Not available	No	Yes	Panel
288	Female	VUS	SDHB	c.307A>G (p.Met103Val)			Yes	No	No	No	No	No	≤45	Kidney	NA	Yes	Yes	Panel
289	Female	Positive	BRCA1	c.3331_3334	Deletion	Frameshift	Yes	Yes	No	No	No	No	46-60	No	Not available	Yes	Yes	Panel
290	Female	VUS	MSH3	c.1647G>C (p.Gln549His)			Yes	Yes	No	No	No	No	≤ 45	No	Triple positive	No	Yes	Panel
291	Female	VUS	RET	c.2432C>G(p.S811C)			Yes	Yes	No	No	No	No	≤ 45	No	Triple positive	Yes	Yes	Panel
292	Female	VUS	RAD51D	c.192C>T(p.F64F)			Yes	Yes	No	No	No	No	≤ 45	No	Not available	Yes	Yes	Panel
293	Female	Negative	NA	NA			Yes	Yes	No	No	No	No	≤ 45	No	Not available	Yes	Yes	Panel
294	Female	Negative	NA	NA			Yes	Yes	No	No	No	No	≤ 45	No	Not available	No	Yes	Panel
295	Female	Negative	NA	NA			Yes	Yes	No	No	No	No	46-60	No	Not available	Yes	Yes	Panel
296	Female	Negative	NA	NA			Yes	Yes	No	No	No	No	46-60	No	Not available	No	Yes	Panel
297	Female	VUS	CHEK2	c.1510G>C (p.Glu504Gln)			Yes	Yes	No	No	No	No	≤ 45	No	ER+, PR+, HER2−	No	Yes	Panel
298	Female	Negative	NA	NA			Yes	Yes	No	No	No	No	≤ 45	No	Not available	Yes	Yes	Panel
299	Female	VUS	MLH1	crom 3 posição 37042550			Yes	No	No	Yes	No	No	46-60	No	NA	Yes	Yes	Panel
300	Female	Negative	NA	NA			Yes	Yes	No	No	No	No	>60	No	ER+, PR-, HER2−	Yes	No	Panel
301	Female	Negative	NA	NA			Yes	Yes	No	No	No	No	46-60	No	ER+, PR+, HER2−	No	Yes	Panel
302	Female	VUS	BARD1	c.1912G>C (p.Ala638Pro)			No	No	No	No	No	No	46-60	No	NA	Yes	Yes	Panel
303	Female	VUS	BAP1	c.1166G>A (p.Arg389His)			Yes	Yes	No	No	No	Yes	46-60	Kidney	ER+, PR+, HER2−	Yes	Yes	Panel
304	Male	Positive	PALB2	c.3426dup	Duplication	Frameshift	No	No	No	No	No	No	>60	No	NA	Yes	Yes	Panel
305	Female	VUS	RET	c.2607+4C>T (Intronic)			Yes	Yes	No	No	No	No	46-60	No	Not available	Yes	No	Panel
306	Female	Negative	NA	NA			Yes	Yes	No	No	No	No	≤ 45	Thyroid	Not available	Yes	Yes	Panel
307	Female	Negative	NA	NA			Yes	Yes	No	No	No	No	46-60	No	Not available	Yes	Yes	Panel
308	Female	VUS	BRCA2	c.8098A>G (p.Ile2700Val			Yes	Yes	No	No	No	No	46-60	No	Triple positive	Yes	Yes	Panel
309	Female	VUS	CDKN1B	c.125C>T (p.Thr42Ile)			Yes	Yes	No	No	No	No	≤ 45	No	ER+, PR+, HER2−	Yes	Yes	Panel
310	Female	VUS	BLM	c.3427G>A (p.Glu1143Lys)			Yes	Yes	No	No	No	No	≤ 45	No	ER+, PR+, HER2−	No	Yes	Panel
311	Male	VUS	PDGFRA	c.1283C>G (p.Thr428Ser)			Yes	No	No	No	No	No	46-60	Prostate	NA	Yes	Yes	Panel
312	Female	Negative	NA	NA			No	No	No	No	No	No	≤45	No	NA	Yes	Yes	Panel
313	Female	Negative	NA	NA			No	No	No	No	No	No	46-60	No	NA	Yes	Yes	Panel
314	Female	VUS	RET	c.2166G>T (p.Lys722Asn)			No	No	No	No	No	No	≤45	No	NA	Yes	Yes	Panel
315	Male	VUS	ECQL4	c.136C>A (p.Arg46Ser)			No	No	No	No	No	No	46-60	No	NA	Yes	Yes	Panel
316	Male	Positive	BRCA1	c.441+2T>A	Single nucleotid	Splice donor	No	No	No	No	No	No	>60	No	NA	Yes	Yes	Panel
317	Female	Positive	BRCA2	c.4005dup	Duplication	Frameshift	Yes	Yes	No	No	No	No	≤ 45	No	Not available	Yes	Yes	Panel
318	Male	Positive	BRCA1	c.3331_3334	Deletion	Frameshift	No	No	No	No	No	No	≤45	No	NA	Yes	Yes	Panel
319	Female	Negative	NA	NA			Yes	Yes	No	No	No	Yes	> 60	GIST	ER+, PR+, HER2−	Yes	Yes	Panel
320	Male	VUS	BRIP1	c.588C>G (p.Asn196Lys)			Yes	No	No	No	No	No	46-60	Prostate	NA	Yes	Yes	Panel
321	Female	Negative	NA	NA			No	No	No	No	No	No	46-60	No	NA	Yes	Yes	Panel
322	Female	Positive	BARD1	c.2279C>A	Single nucleotid	Nonsense	Yes	Yes	No	No	No	No	≤ 45	No	Triplo Negative	No	Yes	Panel
323	Female	Positive	BRCA1	c.3331_3334	Deletion	Frameshift	No	No	No	No	No	No	≤45	No	NA	Yes	Yes	Panel
324	Female	VUS	TCH1	c.646A>G (p.Met216Val			Yes	Yes	No	No	No	No	≤ 45	No	Triple positive	Yes	Yes	Panel
325	Female	Negative	NA	NA			No	No	No	No	No	No	≤45	No	NA	Yes	Yes	Panel
326	Female	VUS	NTHL1	c.704G>A (p.Gly235Asp)			Yes	Yes	No	No	No	No	≤ 45	No	ER+, PR+, HER2−	Yes	Yes	Panel
327	Female	VUS	BRCA2	c.6586A>G (p.Lys2196Glu)			Yes	Yes	No	No	No	No	46-60	No	ER+, PR+, HER2−	Yes	Yes	Panel
328	Female	Negative	NA	NA			Yes	Yes	No	No	No	No	≤ 45	No	Triple negative	Yes	Yes	Panel
329	Female	Negative	NA	NA			No	No	No	No	No	No	≤45	No	NA	Yes	Yes	Panel
330	Female	Positive	BRCA1	c.791_794del	Deletion	Frameshift	Yes	No	No	Yes	No	No	> 60	No	NA	Yes	Yes	Panel

ID	Sex Female or Male	Result	Gene	Description (MANE SELECT)	Variant type	Molecular consequence	Personal Cancer Diagnosis Yes (Affected); No (Not Affected)	Breast Cancer Yes; No	Bilateral Cancer Yes; No	Ovarian Cancer Yes; No	Breast and Ovarian Cancer Yes; No	Breast and Other Cancer Yes; No	Age at First Cancer Diagnosis (Breas or Ovarian)<br>≤ 45; 46–60; > 60; Not available	Other cancer Yes (which one); No	Subtypes, Receptor Status<br>ER+, PR+, HER2−<br>ER+, PR-, HER2−<br>ER+, PR−, HER2+<br>ER−, PR−, HER2+<br>Triple positive<br>Triple negative<br>Not available	First/Second degree family member with breast or ovarian cancer Yes; No; Not available	PFirst/Second degree family member with any cancer Yes; No; Not available	Genetic Testing
331	Female	VUS	CDK4	c.905C>T (p.Pro302Leu)			Yes	Yes	No	No	No	No	≤ 45	No	HER2+	Yes	No	Panel
332	Female	Negative	NA	NA			No	No	No	No	No	No	46-60	No	NA	Yes	Yes	Panel
333	Female	Negative	NA	NA			No	No	No	No	No	No	46-60	No	NA	Yes	Yes	Panel
334	Female	Positive	CHEK2	c.846+1G>C	Single nucleotid	Splice donor	Yes	Yes	No	No	No	No	46-60	No	Not available	Yes	Yes	Panel
335	Female	Negative	NA	NA			No	No	No	No	No	No	≤45	No	NA	Yes	Yes	Panel
336	Female	Negative	NA	NA			Yes	Yes	No	No	No	No	46-60	Thyroid	Not available	Yes	Yes	Panel
337	Female	VUS	MEN1	c.188T>A (p.Phe63Tyr)			No	No	No	No	No	No	≤45	No	NA	Yes	Yes	Panel
338	Female	Positive	PALB2	c.355del	Deletion	Frameshift	No	No	No	No	No	No	>60	Pancreas	NA	Yes	Yes	Panel
339	Female	Positive	PALB2	c.1042C>T	Single nucleotid	Nonsense	Yes	Yes	No	No	No	No	46-60	No	Triple negative	No	No	Panel
340	Female	Positive	BRCA2	c.4005dup	Duplication	Frameshift	Yes	Yes	No	No	No	No	≤ 45	No	ER+, PR+, HER2−	Yes	Yes	Panel
341	Female	Negative	NA	NA			No	No	No	No	No	No	46-60	No	NA	Yes	Yes	Panel
342	Male	VUS	APC	c.8524T>G (p.Ser2842Ala)			Yes	No	No	No	No	No	>60	Skin	NA	Yes	Yes	Panel
343	Female	VUS	ALK	c.1948C>A (p.Pro650Thr)			Yes	Yes	No	No	No	No	> 60	CCR	Triple negative	Yes	Yes	Panel
344	Female	Negative	NA	NA			Yes	Yes	Yes	No	No	No	46-60	No	Not available	Yes	Yes	Panel
345	Female	Negative	NA	NA			Yes	No	No	No	No	No	46-60	Skin	NA	Yes	Yes	Panel
346	Female	VUS	CDKN1C	c.644C>A (p.Ala215Asp)			No	No	No	No	No	No	≤45	No	NA	Yes	Yes	Panel
347	Female	VUS	CDH1	c.2332G>A (p.Ala778Thr)			Yes	Yes	No	No	No	No	> 60	No	Skin	No	Yes	Panel
348	Female	VUS	BRCA2	c.9235G>T p.(Val3079Phe)			Yes	Yes	No	No	No	No	≤ 45	No	Not available	No	Yes	Panel
349	Female	VUS	RET	c.2371T>A (p.Tyr791Asn)			Yes	Yes	No	No	No	No	≤ 45	No	Not available	No	No	Panel
350	Female	Positive	BRCA2	c.9382C>T	Single nucleotid	Nonsense	Yes	Yes	No	No	No	No	≤ 45	No	ER+, PR+, HER2−	Yes	Yes	Panel
351	Female	Positive	BRCA1	c.3331_3334	Deletion	Frameshift	Yes	Yes	Yes	No	No	No	≤ 45	No	Not available	Yes	Yes	Panel
352	Female	Positive	BRCA1	c.5074+2T>C	Single nucleotid	Splice donor	Yes	Yes	No	Yes	Yes	No	≤ 45	No	Triple negative	Yes	Yes	Panel
353	Female	Positive	BRCA2	c.4808del	Deletion	Frameshift	Yes	Yes	No	No	No	Yes	≤ 45	Thyroid	Not available	Yes	Yes	Panel
354	Female	Negative	NA	NA			Yes	Yes	No	No	No	No	> 60	No	ER+, PR+, HER2−	Yes	Yes	Panel
355	Female	Positive	BRCA1	c.3331_3334	Deletion	Frameshift	Yes	Yes	No	Yes	Yes	No	> 60	No	ER-, PR+, HER2−	Yes	No	Panel

ID
1
2
3
4
5
6
7
8
9
10
11
12
13
14
15
16
17
18
19
20
21
22
23
24
25
26
27
28
29
30
31
32
33
34
35
36
37
38
39
40
41
42
43
44

ID
45
46
47
48
49
50
51
52
53
54
55
56
57
58
59
60
61
62
63
64
65
66
67
68
69
70
71
72
73
74
75
76
77
78
79
80
81
82
83
84
85
86
87
88
89
90
91
92

ID
93
94
95
96
97
98
99
100
101
102
103
104
105
106
107
108
109
110
111
112
113
114
115
116
117
118
119
120
121
122
123
124
125
126
127
128
129
130
131
132
133
134
135
136
137
138
139
140

ID
141
142
143
144
145
146
147
148
149
150
151
152
153
154
155
156
157
158
159
160
161
162
163
164
165
166
167
168
169
170
171
172
173
174
175
176
177
178
179
180
181
182
183
184
185
186

ID
187
188
189
190
191
192
193
194
195
196
197
198
199
200
201
202
203
204
205
206
207
208
209
210
211
212
213
214
215
216
217
218
219
220
221
222
223
224
225
226
227
228
229
230
231
232
233
234

ID
235
236
237
238
239
240
241
242
243
244
245
246
247
248
249
250
251
252
253
254
255
256
257
258
259
260
261
262
263
264
265
266
267
268
269
270
271
272
273
274
275
276
277
278
279
280
281
282

ID
283
284
285
286
287
288
289
290
291
292
293
294
295
296
297
298
299
300
301
302
303
304
305
306
307
308
309
310
311
312
313
314
315
316
317
318
319
320
321
322
323
324
325
326
327
328
329
330

ID
**331**
**332**
**333**
**334**
**335**
**336**
**337**
**338**
**339**
**340**
**341**
**342**
**343**
**344**
**345**
**346**
**347**
**348**
**349**
**350**
**351**
**352**
**353**
**354**
**355**
