# Supplementary material for: Hereditary Breast Cancer in the Brazilian State of Ceará (The CHANCE Cohort): Higher-Than-Expected Prevalence of Recurrent Germline Pathogenic Variants
Source: Front Oncol. 2022 Jul 22;12:932957. doi: 10.3389/fonc.2022.932957 (PMC9361024; doi:10.3389/fonc.2022.932957)
Supplement: Supplementary file 2 [file Table_1.docx]

| Supplementary Table 1. Variants of Uncertain Significance: Mutation spectrum and frequency. | | |
| --- | --- | --- |
| Gene | Number of Patients | VUS |
| *AIP* | 1 | c.965C>T |
| *ALK* | 1  1 | c.1948C>A  c.3254C>A |
| *APC* | 1  1  1  1  1 | Crom 5*  c.1580G>C  c.6637A>G  c.6782C>T  c.30366C>T |
|  | 2 | c.8524T>G |
| *ATM* | 1  1  1  1  1  1  1 | c.1049C>T  c.4148C>T  c.4424T>G  c.5753G>C  c.6998C>T  c.7816A>G  c. 7375C>G |
| *AXIN2* | 1  1 | c.1577A>G  c.1829G>A |
| *BAP1* | 1 | c.1166G>A |
| *BARD1* | 1  1 | c.1912G>C  c.2206T>C |
|  | 2 | c.1835A>T |
| *BLM* | 1  1  1  1 | c.1216A>G  c.1601A>G  c.2686C>T  c.3427G>A |
|  | 2 | c. 543C>T |
| *BRIP1* | 1  1  1 | c.588C>G  c.797C>T  c.1066C>G |
| *BRCA1* | 1  1  1  1  1 | c.1012A>G  c.1114A>C  c.1724A>G  c.4097-141A>C  c.5580C>G |
| *BRCA2* | 1  1  1  1  1  1  1  1  1  1  1  1  1  1  1  1  1  1  1 | c.7A>G:  c.175C>A  c.383A>G  c.1114A>C  c.1148T>A  c.1483G>A  c.2401A>G  c.4091A>G  c.4097-141A>C  c.5270A>G  c.6586A>G  c.7676C>G  c.8094A>G  c.8098A>G  c.9235G>T  c.9940A>G  c.9976A>T  c.A1114C  c.G5704A |
|  | 2 | c.4187A>G |
| *CASP8* | 1 | c.35A>C |
| *CASR* | 1 | c.856C>T |
| *CDH1* | 1  1 | c.68A>T  c.2232G>A |
| *CDK4* | 1 | c.905C>T |
| *CDKN1B* | 1  1 | c.125C>T  c.349C>T |
| *CDKN1C* | 1  1  1 | c.389A>T  c.644C>A  c.1510G>C |
| *CDKN2A* | 1 | c.9_32del |
| *CHEK2* | 1  1  1  1 | c.349C>T  c.1130A>G  c.1141A>G  c.1510G>C |
| *CTNNA1* | 1  1  1 | c.710A>G  c.1070G>A  c.2191C>T |
| *DIS3L2* | 1  1  1 | c.1447C>G  c.1687G>A  c.2227C>T |
| *ECQL4* | 1 | c.136C>A |
| *EGFR* | 1 | c.2885G>A |
| *FLCN* | 1 | c.65C>T |
| *GATA2* | 1 | c.797T>C |
| *HRAS* | 1 | c.290+6C>A |
| *KIT* | 1 | c.1853T>C |
| *MEN1* | 1  1  1 | c.188T>A  c.883C>T  c.3128C>T |
| *MLH1* | 1  1  1 | c.5C>T  c.1270G>A  Chr3 37042550 C>T |
| *MSH2* | 1  1  1 | c.314A>G  c.703A>G  Chr2 47637301 T>G |
| *MSH3* | 1  1  1  1 | c.205C>G  c.1647G>C  c.2623G>A  Del (exons9-12) |
| *MSH6* | 1  2 | c.175C>A  c.1730G>A |
| *MUTYH* | 1  1  1 | c.650G>A  c.667A>G  c.1309C>T |
| *NF1* | 1  1 | c.100G>A  c.7150A>G |
| *NF2* | 1 | c.243_248del |
| *NTHL1* | 1 | c.704G>A |
| *PALB2* | 1  1 | c.1217C>T  c.2773G>C |
| *PDGFRA* | 1  1  1 | c.1283C>G  c.1438G>A  c.3216G>T |
| *PMS2* | 1  1 | c.932A>G  c.1561G>A |
| *POLD1* | 1 | c.353C>T |
| *POLE* | 1  1  1  1 | c.-6G>C  c.158T>C  c.577A>G  c.3857G>A |
| *PTCH1* | 1 | c.4202A>G |
| *RAD50* | 1 | c.610A>G |
| *RAD51C* | 1 | c.904G>A |
| *RAD51D* | 1  1 | c.109del  c.192C>T |
| *RET* | 1  1  1  1 | c.2432C>G  c.2166G>T  c.2371T>A  c.2607+4C>T |
| *SDHB* | 1  1 | c.112C>T  c.307A>G |
| *SDHC* | 1 | c.5C>G |
| *STK11* | 1  1 | c.631C>T  c.1108G>C |
| *TCH1* | 1 | c.646A>G |
| *TERT* | 1 | c.1336C>G |
| *TSC2* | 1  1 | c.2048C>A  c.3779C>T |
| *WRN* | 1  1 | c.58A>G  c.2300C>G |
